# Gender (in)equality in divorce in Switzerland: Lawyers’ formal-egalitarian vs. compensatory interpretations

**DOI:** 10.1080/09649069.2025.2454103

**Published:** 2025-02-12

**Authors:** Gaëlle Aeby, Michelle Cottier, Eric D. Widmer, Bindu Sahdeva

**Affiliations:** a School of Social Work, HES-SO Valais-Wallis, Sierre, Switzerland; b Centre for Legislative Studies, University of Geneva, Geneva, Switzerland; c Institute of Sociological Research & Centre for Legislative Studies, University of Geneva, Geneva, Switzerland

**Keywords:** Divorce law, lawyers, maintenance, pension, SDG 5: Gender equality

## Abstract

While family law in Western countries has changed dramatically in recent decades towards the ideal of gender equality, many inequalities remain with regard to the gendered outcomes of separation and divorce. Moreover, in family law, the move towards private ordering of legal issues in divorce has shifted the focus from the courts to lawyers, who have become central to the negotiation process in and out of court. Consequently, the interpretation of the law by lawyers needs to be scrutinised in order to assess its potential impact on the outcome of divorce settlements in terms of gender equality. Based on a large-scale survey of Swiss divorce lawyers and using a scenario approach, we examine the division of pension assets and post-marital maintenance, issues that are critical to the long-term socio-economic consequences of the unequal division of paid and unpaid work between couples. From an empirical perspective, the article examines the preferences of divorce lawyers with respect to the *formal-egalitarian*, *compensatory* and *traditionalist* interpretations. The findings suggest that the *formal-egalitarian* interpretation of gender equality is gaining traction in Switzerland, and that an awareness of gender inequalities is necessary in order to make full use of the compensatory mechanisms embedded in written law.

## Introduction

While family law in Western countries has changed dramatically in recent decades, moving towards the ideal of gender equality and equal treatment of all family members, many inequalities remain, particularly with regard to the gendered outcomes of separation and divorce for different-sex couples (Bessière *et al*. 2013, Bastard *et al*. 2016, Fisher and Low 2016, 2018). In particular, there is a tension between the autonomy principle and the compensation principle, which is anchored in the distinction between formal and substantive equality (Fredman 2016). The autonomy principle aims to achieve economic independence for both ex-spouses after divorce based on a *formal-egalitarian* interpretation of gender equality, while the compensation principle seeks to offset the long-term disadvantages of the unequal distribution of paid and unpaid work between male and female spouses during the marriage, even after its end based on a *compensatory* interpretation of gender equality. In the background of this balancing act between autonomy and compensation, another interpretation remains entrenched, the *traditionalist* one, according to which the main male breadwinner is entitled to more because of a higher value given to paid work (Binkert and Wyss 1997).

In Western countries with a liberal orientation to both private family law and the welfare state, such as the UK and Switzerland, the autonomy principle is gaining ground, despite the persistence of empirically documented inequalities (Douglas 2018, Kessler 2020). In addition, ‘fair’ compensation tends to be difficult to determine legally and socially (Scherpe 2016). Indeed, three components of the financial consequences of divorce need to be considered in a settlement: maintenance in relation to income, property, and pension assets. While property in the form of home ownership is a key issue in the UK, with around 61.7% of households being home owners (House of Commons 2024), the situation is different in Switzerland, where home ownership is less common, with only 35% of the population owning their home (Federal Statistical Office 2024). Pensions are a key issue in Swiss divorces, alongside maintenance, as they are the form of savings the greatest number of people have.

The principles of autonomy and compensation may come into conflict when dividing income, property and pension assets. As a result, it may increase the risk of unequal divorce outcomes in terms of substantive equality between ex-spouses in Switzerland. It has become a matter of concern not only in terms of its normative foundations in private family law, but also for the welfare state system, which in the event of inadequate provision ends up providing social assistance to the weaker parties, often single mothers and their children, with often detrimental consequences in terms of reputation and further debts to be partially reimbursed by the receiving party to the state as social assistance is not free in Switzerland (Bonvin *et al*. 2020).

At the same time as this strengthening of the principle of autonomy, since the 1970s there has been a move towards private ordering of the legal issues of separation and divorce (Maclean *et al*. 2015). Switzerland adopted this trend later than the UK, but since the 2000 divorce reform (Federal Council 1996, Schwenzer 2011), the family justice system has tended to encourage more divorcing couples to reach agreement on all aspects of their divorce (Cottier *et al*. 2023). On the one hand, the responsibility for negotiating a divorce agreement was shifted onto the shoulders of the divorcing parties, who often lack adequate legal knowledge (Woodward and Sefton 2014, Hitchings *et al*. 2023). On the other hand, it strengthened the role of divorce lawyers, as they are at the centre of the negotiation process in and out of court. The prevalence of using a lawyer varies between countries: in England and Wales, only 32% of divorcees were found to have used legal services in relation to their financial arrangements (Hitchings *et al*. 2023), while in Switzerland judges reported that only 29% of settlements were made in the complete absence of a lawyer (Federal Office of Justice 2005), which may be partly explained by the availability of legal aid for those on low incomes.

The different actors who apply the law are part of an ongoing process of legal intermediation that contributes to the shaping of legal norms, and thus they participate in defining how gender equality is interpreted and implemented (Barlow *et al*. 2017, Pélisse 2019, Friedli 2021, Cottier *et al*. 2023). The assistance of a lawyer may lead to quite different outcomes in terms of gender equality, as legal frameworks usually leave room for interpretation (Binkert and Wyss 1997). Lawyers have different views of their role – for instance, some are more ‘legal-craft’ or ‘client-adjustment’ oriented (Mather *et al*. 2001), but to our knowledge, the issue of the influence of their personal experiences and attitudes on the outcome of a divorce negotiation has not been explored to date, possibly because of the assumption that they will always seek the best outcome for their client.

This research examines the preferences of Swiss divorce lawyers with regard to the *formal-egalitarian*, *compensatory* and *traditionalist* interpretations from an empirical perspective. We first provide an overview of the state of research on gender (in)equality and the private settlement of divorce consequences and analyse the current legal framework in Switzerland regarding the division of pension assets and post-marital maintenance which are of particular interest given the possibility for lawyers to advocate for one or the other interpretation. In a scenario approach, we then use a survey of divorce lawyers to explore the lawyers’ interpretations and assess their association with the lawyers’ socio-demographic characteristics, their professional and personal experiences, and their attitudes towards equality principles and gender norms. Finally, we draw conclusions and discuss the issue of gender equality after divorce and the socio-economic consequences for the economically weaker party and children.

### Gender (In)equality in society and the functions of divorce law

In family law in Western countries, socio-legal studies have described a move towards private ordering of the legal issues of separation and divorce since the 1970s (Maclean *et al*. 2015), shifting the focus from courts to lawyers and other professionals (mediators, family therapists, social workers etc.) assisting separating families (Biland 2023), and to divorcing parties themselves (Hitchings *et al*. 2023). When involved, lawyers play a key role in the negotiation process happening ‘in the shadow of the law’ (Mnookin and Kornhauser 1978–79). However, the legal framework and its interpretations by lawyers are embedded in a wider societal context in which the sharing of paid and unpaid work responsibilities between men and women has become one of the challenges facing Western countries in the 21st century (Salin *et al*. 2018). Indeed, while the participation of women, particularly mothers, in the labour market has increased (European Union 2017), the involvement of fathers in unpaid care work has not kept pace (Esping-Andersen 2009, Salin *et al*. 2018). This translates into a weakening of the traditional ‘male breadwinner and female caregiver’ model, and its replacement by hybrid models highly dependent on labour market institutions and family-oriented policies (Cipollone *et al*. 2014). Switzerland is an excellent case study of the lack of adequate family policies, as evidenced, for example, by the expenditure on family/children benefits, for which Switzerland allocates only 1.6% of GDP in 2021 (EU average: 2.4%) (Eurostat 2024a), or the late introduction in 2021 of a short two-week paternity leave coupled with a 14-week maternity leave – both only corresponding to the minimum recommended by EU legislation on work-life balance (Jurviste and Lecerf 2022) – without an accompanying parental leave. This pushes a majority of married different-sex couples to opt for a ‘semi-traditional’ family arrangement in which the wife is the primary caregiver and works part-time, while the husband is the main breadwinner (Maihofer 2014, Le Goff and Levy 2016), a model similar to that prevailing in the UK (Lyonette 2015). However, marriage is more common in Switzerland for couples with children, as shown by the 26% birth rate outside marriage in 2018 (compared to 48% in the UK) (Eurostat 2020). In European comparison, Switzerland recorded one of the highest shares of women working part-time, 60% of total employment against 19% for men, just below the Netherlands but with the largest difference between women and men (41% points) (Eurostat 2024b). This translates into clear income differences between women and men, with the gender overall earnings gap (GOEG) reaching 43.2% in 2018 and the gender pension gap reaching 34.6% in 2020 (Federal Council 2022). Interestingly, this ‘semi-traditional’ model coexists with egalitarian gender values that are present before and persist after the birth of children, despite the shift to an unequal work-family organisation after the transition to parenthood, as in other liberal welfare states (Bühlmann *et al*. 2010). In the event of divorce, the division of labour practiced during the marriage is in most cases continued. That means that, on the one hand, a majority of divorced fathers are at greater risk of seeing their children less often, as it happens elsewhere (Kalmijn 2015). On the other hand, a majority of divorced mothers have to shoulder day-to-day child-rearing responsibilities alone, which puts them into a difficult position in relation to labour market participation, economic security, old age provision and health (Levy and Widmer 2013, Cottier *et al*. 2017). Overall, a large number of studies have documented the economic consequences of divorce depending on gender and presence of children and showed that mothers are more likely to suffer significant drops in household income and to fall into poverty (Fisher and Low 2016, 2018, Hogendoorn *et al*. 2019, Leopold and Kalmijn 2016). For divorce law, the question is, to what extent the disadvantages ensuing from the unequal division of paid and unpaid labour during the marriage and after divorce are compensated through post-marital maintenance, the division of property and pension assets. The answer to this question will strongly depend on how the functions of divorce law in relation to gender equality are defined and implemented in lawyers’ strategies and their consequences in actual court decisions.

The financial consequences of divorce are regulated in very different ways amongst Western jurisdictions. The main difference is between the common law jurisdictions which provide for the financial consequences of divorce as a package, and the continental European jurisdictions of the civilian tradition, like Switzerland, where the law distinguishes between different ‘pillars’ such as the division of matrimonial property, division of pension assets, maintenance, or allocation of use of the family home (Scherpe 2016). In addition, to get a finer picture, the type of welfare state must be considered, as national family policy responses across countries in Europe are quite contrasted, with liberal welfare states such as the UK and Switzerland emphasising individual responsibility the most (Esping-Andersen 2009). Three overarching regulatory trends in family law can be identified: sharing but only the fruits of joint labour; covering the needs but with time limits to post-marital maintenance claim, and compensating to a certain extent the ‘relationship generated losses’ of the one spouse, usually a woman, who has invested in family life because such investments are beneficial to society (Scherpe 2016). However, the ’what’ and ’how’ of compensation is difficult to determine.

Moreover, the legislative framework generally leaves some room for interpretation, which practitioners will use differently. Binkert and Wyss (1997) showed that interpretations of gender equality regarding post-marital maintenance vary strongly in the practice of Swiss first instance courts’ judges. Three interpretations were identified: The *formal-egalitarian* interpretation is based on the desire for economic independence for both ex-spouses after divorce and the principle of autonomy. It requires that the wife quickly achieves financial independence and it ignores the reality of the lasting unequal division of childcare responsibilities after a divorce while resulting in a low level of post-marital maintenance payments (Binkert and Wyss 1997, p. 302). In contrast, the *compensatory* interpretation aims to counterbalance the lasting negative consequences of the unequal distribution of paid and unpaid work even after the divorce. As it defines mothers as the primary caregivers who must be compensated for their care work through post-marital maintenance payments, it has the disadvantage of entrenching the previous division of roles beyond the end of the marriage (Binkert and Wyss 1997, pp. 94–95). Finally, the authors also identified a *traditionalist* interpretation which sees the division of labour based on gender as natural. According to this interpretation, the husband’s economically privileged position is not to be challenged and his interests should generally prevail. Furthermore, it assumes that the most effective way for women to achieve economic sustainability is to remarry, and thereby reverting to the male breadwinner model (Binkert and Wyss 1997, p. 302). This interpretation results into very low post-marital maintenance payments.

Looking at recent trends, it appears that the *formal-egalitarian* interpretation is gaining traction. There has been a significant loss of post-marital maintenance after divorce between 1990 and 2008 in Switzerland and a systematic study concluded that the increase in Swiss women’s economic self-sufficiency has not kept pace with the decline in maintenance payments, i.e. that the decrease in maintenance payments has on average led to a lower income for divorced women (Kessler 2020). The ever-increasing dominance of the *formal-egalitarian* interpretation in written divorce law may be one explanation for this long-term development, which is economically disadvantageous for women who have partially withdrawn from the labour market to raise their children. In a recent study nine decisions of the Swiss Federal Supreme Court between 2006 and 2018 were examined and it was found that in many cases arguments made by the legal literature in favour of a *compensatory* interpretation of post-marital and child maintenance were listed, but a less compensatory direction was chosen for the final decision (Cottier *et al*. 2022). Moreover, recent trends comparing cohorts based on the Swiss Household Panel data between 2000 and 2017 suggest that Swiss millennials are less supportive of measures to promote women in general and believe that women are decreasingly penalised (Bornatici *et al*. 2020). This can be explained by the change in the discourse on gender equality policy since 2000, which has resulted in limited measures and progress in achieving gender equality (Lanfranconi and Valarino 2014).

In the context of the negotiation of divorce settlements, gender-specific behaviours could be another possible explanatory factor for these unequal outcomes. Some authors observe that women negotiate to obtain an apology from their ex-spouse or to achieve stability, and focus on maintaining good relationships rather than maximising their self-interest (Wilkinson-Ryan and Small 2008, Rebouché 2016). At the same time, other authors highlight the danger of reproducing gender stereotypes in research when unfair outcomes in divorce negotiations are accounted for exclusively by stereotypical ‘female’ and ‘male’ negotiating behaviour (Kolb 2012). We share this reluctance to accept gendered negotiation styles as the sole explanation and suggest that the influence of gender on the negotiation process in the context of separation and divorce is related with ‘institutional doing gender’ (Krüger and Levy 2001, Levy and Widmer 2013), in the sense that notions of gender equality present in family law institutions, lawyers’ interpretations and ex-spouses’ life experiences all come into play in shaping divorce agreements.

### Gender (In)equality and the role of divorce lawyers

As stated above, the move towards private ordering of the legal issues of separation and divorce has brought lawyers up to the front line as a new key actor in resolving family disputes. In this context, lawyers’ interpretations of gender equality may have an important impact on the outcome of a divorce settlement in terms of gender equality. A recent large-scale study of family dispute resolution in England and Wales found that lawyers are not always up to the task of achieving an agreement that is meeting needs, compensating for relationship-generated disadvantages and equal sharing of the consequences of the division of labour between the breadwinner and the caregiver (Barlow *et al*. 2017). The study raised concerns about two types of cases: cases where mediators and lawyers fail to provide women with sufficient information, advice, protection, support or representation to enable them to attain an even financial settlement; and cases where women agree to formal equality as a compromise, to their detriment and that of their children, in circumstances where the practitioner could have intervened to prevent such an outcome (Barlow *et al*. 2017). In continental European jurisdictions such as Switzerland, one might think that this risk could be remedied in court, but research has shown that courts are reluctant to refuse to approve a divorce agreement if the parties have been assisted by a lawyer, partly on the grounds of procedural efficiency (Le Collectif Onze 2013, p. 51, Bastard *et al*. 2015, p. 281).

Lawyers play a key role in gathering family stories, standardising and classifying them in order to fit them into the normative framework of the law (Macfarlane 2008, Bastard *et al*. 2015, Barlow *et al*. 2017, Bessière *et al*. 2020, Biland 2023). From this point of view, legal acts of standardisation and classification ratify certain norms of social life, considered legitimate, while concealing others (Flint and Rowlands 2003). The role of lawyers in this respect is primarily tactical, as they have to ‘filter’ the family stories of their clients to produce the divorce agreement, an output that is standardised according to the requirements of divorce proceedings. Lawyers guide their clients and editorialise their requests so that they fit into the normative framework of the law (Bessière *et al*. 2020). At the same time, moral normalisation takes place, again with a strategic aim, so that the lawyer pursues the objective of clarifying the client’s situation in the light of moral conceptions to which compliance is expected, including with regard to the gendered expectations of the legal institution regarding mothers’ and fathers’ roles, meaning the moral standards presupposed by judges, i.e. those of the middle and upper classes (Bessière *et al*. 2020).

Based on the central role of lawyers in the interpretation of gender equality as well as for the presentation of their client’s situation, the question arises as to the existence of different perspectives among different categories of lawyers. Existing research has examined how different professional orientations relate to a lawyer’s gender. Findings suggest that female lawyers tend to place more emphasis on ‘sensitive listening to clients’ (Mather *et al*. 2001), and to use mediation more often than their male counterparts. However, other research has shown that there is no such thing as a gender-specific style of professional behaviour (Bogoch 1997). Similar non-conclusive results were found for family law judges (Bessière and Mille 2013). Thus, the association between gendered interpretations of the law and law practitioners’ gender is yet to be demonstrated.

Overall, lawyers’ interpretation of gender equality and their presentation of their client’s situation may have an important impact in terms of the outcome of the negotiation. The question remains as to whether there exist different perspectives among different categories of lawyers in this regard.

### Gender (In)equality: division of pension assets and post-marital maintenance in Switzerland

In Switzerland, amicable divorce is the ‘regular’ ground for divorce, as estimates suggest that about 90% of all divorces are based on a full agreement (Federal Statistical Office 2010). The agreement must cover child matters (parental responsibility, physical custody, visitation rights, child maintenance), matrimonial property matters, the allocation of the use of the family home, the division of pension assets accrued during marriage, and finally post-marital maintenance (Gloor 2022: N 4;, Leuba *et al*. 2021, pp. 5–7). In Switzerland, every divorce involves a court, which must review and ratify the divorce agreement.^1^ Not all divorces are assisted by a lawyer (Federal Office of Justice 2005), but when they are, the agreement is more likely to be ratified by the court, as has been described for other countries as well (Le Collectif Onze 2013, p. 51, Bastard *et al*. 2015, p. 281), underlining the key role of lawyers. In particular two issues are of importance when speaking of the scope for action of lawyers in terms of gender (in)equality: division of pension assets and post-marital maintenance.^2^

#### Division of pension assets: deviation from the rule of equal sharing

In the year 2000, the fundamental reform of Swiss divorce law which brought about no fault divorce also introduced the principle of the equal sharing of all pension assets accrued by both spouses during marriage. This meant that upon divorce in most cases a part of the husband’s pension assets (which in most Swiss divorces constitute the main savings) are transferred to the wife’s pension fund, thereby equalising the capital accrued during the marriage. This novelty aimed at improving the economic situation of divorced women at the time of retirement age (Federal Council 1996, p. 99). The law was revised in 2017 to allow for more flexibility in applying the rule of equal sharing of accrued pension assets (Federal Council 2013). Today it is possible to deviate, in an agreement reached by the spouses, from the 50/50 division (art. 122 SCC), if an ‘adequate’ old-age and disability provision is provided (Federal Council 2013, Leuba and Udry 2018, Jungo and Grütter 2022). This norm is interpreted in such a way that the court must refuse to approve a divorce agreement regarding the division of pension assets in particular^3^ if it would lead to one of the former spouses depending on social welfare in old age or in the event of disability.^4^ This high threshold for judicial intervention has the consequence that the division of pension assets, when regulated in a divorce agreement, is open to various interpretations of gender equality, including the *compensatory* interpretation that provides for compensation of disadvantages, by attributing more than half of the pension assets accrued by the two spouses during marriage to the ex-spouse who will continue working part-time after divorce due to childcare duties, but also to the *traditionalist* interpretation that contradicts the original objective of equalising pension assets at the time of divorce.^5^ Thus, the question is under what circumstances lawyers recommend the deviation from 50/50 division of assets and who are the lawyers who make use of this possibility when drafting a divorce agreement.

#### Post-marital maintenance: calculation of the hypothetical income

According to Swiss divorce law, post-marital maintenance payments can be fixed to the benefit of the economically weaker parties, especially the care-giving ex-spouse and children. For the computation of maintenance, not only the incomes actually earned are taken into account, but also a hypothetical income can be considered, especially if the combined incomes do not cover the needs of all family members (Büchler 2022: N 34). Two conditions must be cumulatively fulfilled for this: It must be reasonable and possible to achieve the hypothetical income.^6^ The assessment is made considering professional qualifications, age and state of health, as well as the situation in the labour market. In the context of a divorce with children, the question of a hypothetical income is linked to the expectation of an integration into the labour market for the main caring parent, mostly mothers. The Swiss Federal Court has recently moved towards a quicker reintegration of caregiver parents into the labour market: Older case law expected caregivers to take up a salaried occupation only when the youngest child attained the age of ten. This changed with a landmark case from 2018^7^: a gradual reintegration into the labour market is now expected from the time the youngest child enters compulsory schooling, at around four to six years of age depending on their cantonal schooling system. From this point on, 50% employment is expected. This school level model limits the margin of interpretation for lawyers: the duration of compensation is framed by children’s school age, and only the special needs of a child or the care of a large number of children allow for a later entry into the labour market, a lower hypothetical income and consequently longer and higher maintenance payments. However, when computing the amount of the hypothetical income, room for lawyers to push either for more autonomy or for more compensation remains.

### Research question and hypotheses

Based on the gender interpretation model described above (Binkert and Wyss 1997), we start from the assumption that there are three main normative interpretations of equality that attempt to resolve dilemmas in the divorce context regarding the division of pension assets and post-marital maintenance: *formal-egalitarian, compensatory* and, to a lesser extent, *traditionalist*. However, we make this model more nuanced by hypothesising that interpretations given by lawyers in their practices can be mixed. Divorce lawyers, when negotiating a divorce settlement, may propose solutions to their clients’ dilemmas that are based on their gendered interpretations of the written law. This research examines the influence of a number of factors related to lawyers on normative interpretations: their socio-demographic characteristics, their professional and personal experiences, and their attitudes towards equality principles and gender norms.

## Materials and methods

### Research project

We conducted a 4-year interdisciplinary research project dealing with gender equality in Swiss family law entitled *The negotiation of divorce agreements and gender (in)equality in Switzerland*. The project comprises three parts following a sequential explanatory design: an analysis of written law (1), a survey targeting divorce lawyers (2), and qualitative interviews with lawyers and divorcees (3). The results presented in this article are based on the quantitative survey of a sample of 600 lawyers practicing divorce law in the 26 cantons of Switzerland.

### Data and sample

To be able to construct a large sample of lawyers practicing divorce law in the 26 cantons of Switzerland, contact information was collected from three different free public online sources (Swiss Bar Association; cantonal bar associations; Democratic Lawyers of Switzerland). We obtained a dataset of 1487^8^ valid contacts to whom we sent the self-administered questionnaire, offering different response options to increase participation: We provided online and paper versions in German and French. No incentive was paid. Data collection lasted just over three months, from the end of September 2020 to the beginning of January 2021. The participation rate of 40.55% is considered satisfactory compared to other survey participation rates, especially in the Swiss legal field (van Gestel *et al*. 2018). After a quality check of the data, we obtained a data set of 600 participants. The sample was composed of 56% female lawyers, 43% male lawyers and two lawyers who did not indicate their gender.^9^ All age groups were adequately represented: 23% of respondents were 39 years old or younger, 24% were between 40 and 49 years old, 30% were between 50 and 59 years old and 23% were 60 years old or older.

### A scenario approach

Based on the interpretations of gender equality (*formal-egalitarian*, *compensatory*, *traditionalist*), we created four scenarios and formulated possible solutions that reflect such interpretations of gender equality in divorce law. Each scenario consisted of a negotiation configuration in which the clients disagreed on one main issue. The divorce lawyers had to indicate their preferred solution, choosing between four options that indicated a substantive outcome and a rationale. As noted by researchers using this method (Kuhn and Vuille 2010, Jafarkarimi *et al*. 2016), the main bias lies in the fact that the proposed solutions are ideal-types, and it is therefore possible that lawyers (or judges) have a different attitude towards a fictitious case included in a questionnaire than towards a concrete case with real parties. Nevertheless, the approach is so far the best solution to test such questions in an empirical way. The question was as follows: *‘Which of the four combinations (solution and rationale) seems best to you, from an objective point of view? Please tick the number corresponding to the answer you have chosen’*. For respondents who could not decide, there was a ‘no answer’ option. The solutions proposed in the scenarios alone were legally permissible under Swiss divorce law at the time of the survey, in part corresponding to the default rules and in part deviating from them, thus making use of the leeway open to a private autonomous arrangement.

In all hypothetical configurations, the spouses have children and their marriage can be considered ‘life-shaping’ according to the case law in force at the time of the survey.^10^ It should be noted that we did not include tight financial circumstances, as the lack of resources does not allow flexibility in the choice of solutions. The four scenarios address various equality-related issues in Swiss divorce law, often referring implicitly or explicitly to recent changes in legislation and case law previously explained. In this paper, we focus on two scenarios about the division of pension assets and post-marital maintenance.^11^

### The characteristics of divorce lawyers

The way of applying written law may depend on lawyers’ socio-demographic characteristics, their professional and personal experiences as well as the attitudes they hold towards equality principles and gender norms. To explain the likelihood of choosing one solution over the other, we considered a number of explanatory variables, of four types:

- *Lawyers’ socio-demographic characteristics*: gender (female or male), age (39 or under, between 40 and 59 years old, 60 and older), and linguistic regions of Switzerland (French-speaking part and German speaking-part);
- *Lawyers’ professional experience*: having a training in mediation (yes or no), having a specialised training in family law (yes or no), the proportion of divorce cases in their overall practice (lower than half of the cases; half of the cases and higher);
- *Lawyers’ personal experience*: having had the experience of a divorce themselves (yes or no);
- *Lawyers’ attitudes towards equality principles and gender norms*: Three variables aim to understand how lawyers treat their clients according to gender on a 5-item categorical scale ranging from *completely false* to *completely true* (see distribution Table 1, part A). Regarding the treatment of male and female clients, a clear majority state that they treat them the same way (item: *I treat my female and male clients strictly equally*). The answers to the two following questions indicate an overall stronger concern for father-child relationships (item: *With my male clients, I strongly insist on their rights to a personal relationship with their child(ren) in order to promote their role as a father*) than for the risk of female single-parent poverty (item: *With my female clients, I strongly insist on their rights to avoid economically disadvantageous compromises*). We recoded these three items to contrast the lawyers in total agreement with the statement (*completely true*) with their counterparts less assertive or in disagreement. The last variable addresses the awareness of gender inequalities in the Swiss society (item: *Inequality between men and women is no longer a problem in Switzerland*) with a 5-item categorical scale ranging from *completely agree* to *completely disagree* (see distribution Table 1, Part B).

**Table 1.** Distribution of the attitudes towards equality principles and gender norms, percentages.

*Part A. Completely false to completely true*	*Completely false*	*Somewhat False*	*Neither false nor true*	*Somewhat true*	*Completely true*	NA*	*Total*
I treat my female and male clients strictly equally.	1	5	6	30	59	1	100
With my male clients, I strongly insist on their rights to a personal relationship with their child(ren) in order to promote their role as a father.	1	5	24	38	32	2	100
With my female clients, I strongly insist on their rights to avoid economically disadvantageous compromises.	3	10	21	48	18	1	100
*Part B. Completely agree to completely disagree*	*Completely agree*	*Somewhat agree*	*Nor agree nor disagree*	*Somewhat disagree*	*Completely disagree*	NA*	*Total*
Inequality between men and women is no longer a problem in Switzerland	1	7	15	49	26	2	100

*No answer.

## Results

### Scenario - division of pension assets

The first scenario describes a case where the spouses have agreed on the sole physical custody of the two school-aged children for the mother with extensive visitation rights for the father, in doing so following the same task distribution (of childcare) as during marriage. However, they disagree on the division of pension assets. Two solutions envisage the division of pension assets in half (solutions 1 and 2), one solution proposes the division in excess of half in favour of the wife, who was and is the main caregiver of the children before and after the divorce, suggesting a *compensatory* interpretation (solution 3), and one solution mirrors the excess of half division in favour of the husband, the main breadwinner, which is justified by the fact that the housewife enjoyed a comfortable life while her spouse worked full-time, indicating a *traditionalist* interpretation (solution 4). Solutions 1 and 2 both have a *formal-egalitarian* interpretation, but while solution 1 is a purely *formal-egalitarian* interpretation with a justification based on financial independence, solution 2 is a mixed *formal-egalitarian and traditionalist* interpretation, as the justification is based on gender role division.

SCENARIO | DIVISION OF PENSION ASSETS **Parties:**

– Tania: Has a degree in computer science, has been a housewife since the birth of her first child.

– Carl: Has a degree in computer science, works full time as a computer scientist

**Child(ren):** Two children, 8 and 10 years old

**Situation during the relationship:**

– Duration of the relationship: 10 years

– Middle income family

**Problem:**

– Tania and Carl have agreed on the sole physical custody for Tania with extensive visitation rights for Carl

– Tania will gradually return to working life according to the school level model

– However, Carl and Tania do not agree on the division of pension assets

**Solutions:**

1) Tania will return to a professional activity that will enable her to regain her financial independence. As she has about 20 years left until retirement age, she will be able to build up sufficient pension provision. Therefore, the rule of splitting the pension assets in half applies. *[Formal-egalitarian]*

2) During their time together, Tania was able to devote herself entirely to her home, while Carl worked to provide a comfortable life for his family. Now that this division of roles has come to an end, the rule of splitting the pension assets equally applies. *[Formal-egalitarian and Traditionalist]*

3) Due to rapid changes in the digital world, Tania’s qualifications are no longer up to date. Tania will have to start at the lower end of the salary scale. In order to close the pension gaps created by sole custody after the divorce, it would therefore be preferable to obtain one third of the pension assets for Carl and two thirds for Tania. Carl, for his part, will have sufficient time to build up adequate pension provision due to his comfortable professional position. *[Compensatory]*

4) During their marriage, Tania did not have to work and could stay at home while Carl worked full time to provide a comfortable life for their family. Since it was mainly Carl who built up the pension and Tania has time to build up sufficient pension before retirement age, it would be preferable to obtain one-third of the pension assets for Tania and two-thirds for Carl. *[Traditionalist]*

While all four answers are legally permissible, the legal principle is that of equal division of pension assets, and deviations from this are conceived as exceptions. In our scenario, however, we are dealing with a classic breadwinner-caregiver marriage, which was explicitly considered in the revision of the law on the division of pension assets of 2017 with regard to the possibility of an over-half division.

**Figure 1.**
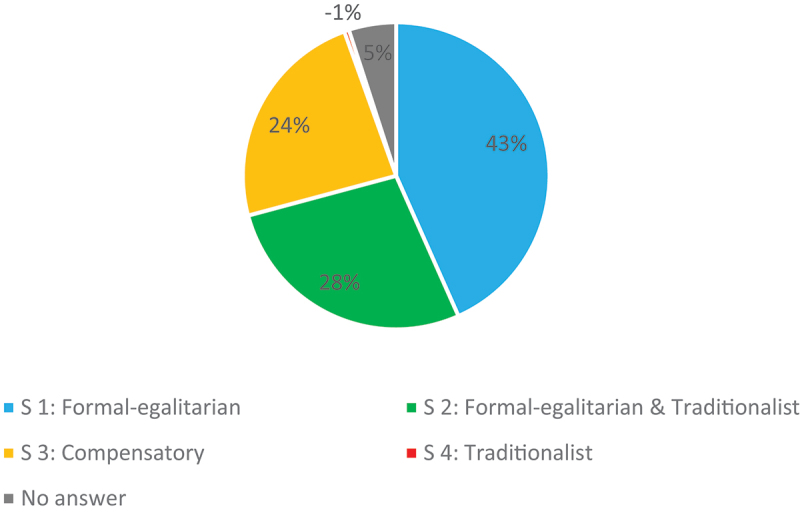
Division of pension assets.

Nevertheless, the two answers that correspond to the legal principle of division in two equal halves, answer 1 and 2, were chosen by most lawyers (see Figure 1). Within these solutions, the purely *formal-egalitarian* interpretation of answer 1 was the most popular overall with 43%, followed by the mixed form of solution 2 with a mixed *formal-egalitarian and traditionalist* interpretation with 28%. The lawyers were rather hesitant to make use of the possibility of an over-half division and thereby compensate for the disadvantages caused by the marital role division also after divorce. Although solution 3 was a textbook example for the application of this possibility introduced by the law of 2017, it was only chosen by 24% of the lawyers. On the other hand, the *traditionalist* interpretation, which grants the working husband an economically privileged position through the under-half split, was chosen by only 1% of the respondents, which indicates that it has largely lost its legitimacy, at least among lawyers working in Switzerland.

We then performed a series of logistical regressions to investigate which variables were associated with choosing the purely *formal-egalitarian* solution, the mixed *formal-egalitarian and traditionalist* solution, and the *compensatory* solution (see Table 2). We did not include the purely *traditionalist* solution as it was chosen by only 1% of the sample. The *compensatory* solution was positively associated with being a female lawyer, and being a lawyer from the German-speaking part of Switzerland. In addition, having a specialised training in family law was also associated with this solution. This solution was also associated with lawyers who strongly believe that gender inequality is still a problem in Switzerland. In contrast, the *formal-egalitarian* solution was associated with being a male lawyer and lawyers believing that gender equality is no longer a problem in Switzerland. Regarding the mixed *formal-egalitarian and traditionalist* solution, only the linguistic region plays a role, as lawyers from the French-speaking part of Switzerland chose this solution more often. Interestingly, reported treatment of clients based on gender did not account for responses given to this scenario.

**Table 2.** Explanatory factors of the interpretations of the scenarios, logistic regressions, odds-ratio.

	Division of assets			Post-marital maintenance: hypothetical income		
	Compensatory	Formel-Egalitarian	Formel-Egalitarian-Traditionalist	Compensatory	Formel-Egalitarian	Compensatory-Traditionalist
***Socio-demographic characteristics of lawyers***						
Female	0.095^*^	−0.108^*^	0.016	−0.045	−0.011	0.067
Age 40–59 *(ref. below 40)*	0.038	−0.075	0.028	−0.105	−0.036	0.133^*^
Age 60 and older *(ref. below 40)*	0.103	0.001	−0.100	−0.122	−0.048	0.157^*^
French-speaking	−0.123^**^	−0.084	0.198***	−0.080	−0.015	0.113^*^
***Professional experiences***						
Training in mediation	−0.008	−0.012	0.015	0.132^**^	−0.044	−0.109^*^
Training in family law	0.115^*^	−0.028	−0.080	0.083	−0.017	−0.074
High proportion of divorce cases	−0.070	0.036	0.034	−0.093	0.025	0.054
***Personal experiences***						
Own divorce	0.017	−0.099	0.078	−0.071	0.033	0.055
***Attitudes toward gender inequality***						
Avoiding female clients’ economic disadvantages	0.093	−0.106	0.011	0.040	−0.055	0.033
Promoting male clients’ fathers’ rights	0.022	−0.010	−0.027	−0.010	0.043	−0.039
Not making any difference based on gender	−0.019	−0.002	0.015	−0.035	−0.003	0.032
Being aware of gender inequalities in society	0.076^***^	−0.060^*^	−0.015	−0.024	−0.035^**^	0.063^**^
Constant	−0.103	0.860***	0.250*	0.687***	0.258***	0.025
Observations	563	563	563	567	567	567
Log Likelihood	−305.163	−392.354	−335.480	−395.600	−64.538	−393.886
Akaike Inf. Crit.	636.326	810.708	696.960	817.200	155.077	813.772

*p**p***p <0.001.

### Scenario – post-marital maintenance: return to work and hypothetical income

The second scenario describes a case where the main care-giving parent returns to work with the issue of what income is to be used to compute maintenance. In this scenario, the maintenance creditor has sole custody of her three-year-old child. According to the school level model of the Swiss Federal Supreme Court (see above 4), she cannot currently be expected to work full-time. Solutions 1 and 2 propose to consider the wife’s last income (before becoming a full-time housewife). This is justified in solution 1 with her skills and the importance of financial independence, which points to a *formal-egalitarian* interpretation. Solution 2 argues that until then the husband has made a greater contribution to the family’s financial maintenance and that it is now up to the wife to increase her contribution, which points to a mixed *formal-egalitarian and traditionalist* interpretation. Solutions 3 and 4 stresses the importance of considering a lower income. In solution 3, the justification is based on the challenges of the labour market and the sharing of the disadvantages of gender roles by both spouses, which is a *compensatory* interpretation, while in solution 4, the justification is based on the importance of maintaining the woman’s role as a mother and avoiding a risk of poverty for her and her child, which can be considered a mixed *compensatory and traditionalist* interpretation.

SCENARIO | POST-MARITAL MAINTENANCE: HYPOTHETICAL INCOME **Parties:**

– Barbara: Chef in a gourmet restaurant until marriage, then housewife

– David: Works full time as an accountant in a SME

**Child(ren)**: One child, 3 years old

**Situation during the relationship:**

– Duration of the relationship: 5 years

– Middle income family

**Problem:**

– Barbara and David have agreed that Barbara will have sole physical custody with extended visitation rights for David.

– Barbara will gradually return to work according to the school level model.

– However, they do not agree on which hypothetical income should be imputed to Barbara.

**Solutions:**

1) The field of catering offers many opportunities and with her qualifications as a chef, it will be possible for Barbara to find a comparable position and gain her independence. Therefore, it would be appropriate to use Barbara’s last salary as a reference for calculating the hypothetical income. *[Formal-egalitarian]*

2) David has provided a comfortable income for the family since marriage and will continue to do so adequately after the divorce. As the workload related to the child will decrease, Barbara should also have to contribute economically to her family with her professional qualifications. Therefore, it would be appropriate to use Barbara’s last salary as a reference for calculating the hypothetical income. *[Formal-egalitarian and Traditionalist]*

3) Barbara and David have opted for a classic division of roles and the resulting consequences should not be borne by Barbara alone. Given the generally known insecurity in the field of catering and her five-year absence, it will be difficult for Barbara to find a job equivalent to the one she held before the marriage. Therefore, it would be better to take this into account when setting a lower hypothetical income. *[Compensatory]*

4) Barbara has mainly taken care of her child and will continue to do so after the divorce. In order to fulfil her role as a mother, she will no longer be able to provide the flexibility required by the catering sector. It is unlikely that Barbara will be able to find a comparably well-paid job, which will put her and her child at risk of poverty. Therefore, it would be better to take this into account when setting a lower hypothetical income. *[Compensatory and Traditionalist]*

**Figure 2.**
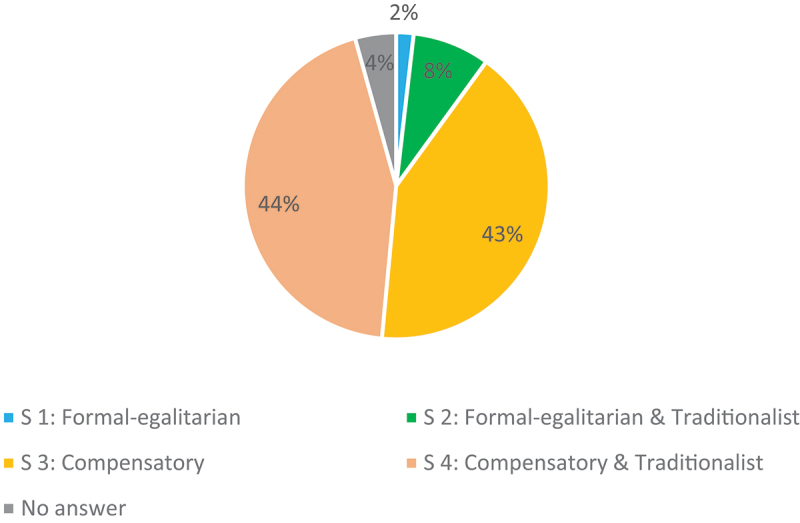
Post-marital maintenance: hypothetical income.

The vast majority of respondents chose solutions 3 and 4, which assume a lower income for the wife than that achieved before marriage (86%) (see Figure 2). This shows the support for *compensatory* arguments in the area of post-marital maintenance: The assumption of a lower hypothetical income compensates for disadvantages that the wife has to face on the labour market due to the five-year absence caused by childcare. However, the lawyers answering the questionnaire did not agree on the justification: 42% of the respondents were in favour of the *compensatory* argumentation 3, which stresses the disadvantages caused by marriage, and 44% were in favour of the mixed *compensatory and traditionalist* solution 4, which argues with the compatibility with the mother’s role. The *formal-egalitarian and traditionalist* interpretation (solution 2), on the other hand, was advocated by only 8% of the sample, and the purely *formal-egalitarian* interpretation (solution 1) by only 2%.

We again performed a series of logistical regressions to investigate which social characteristics of lawyers were associated with choosing the purely *compensatory* solution, the mixed *compensatory and traditionalist*, and the mixed *formal-egalitarian and traditionalist* solution (see Table 2). We did not include the purely *formal-egalitarian* solution as it was chosen by only 2% of the sample. The *compensatory* solution was associated with having a training in mediation. The mixed *compensatory and traditionalist* solution was associated with older lawyers, lawyers from the French-speaking part of Switzerland, with lawyers not having a mediation training, and with lawyers believing that gender equality is still a problem in Switzerland. The mixed *formal-egalitarian and traditionalist* solution was associated with the strong belief that gender equality is no longer a problem in Switzerland. It should be highlighted that lawyers’ gender did not matter for this scenario. Interestingly, as for scenario 1, reported treatment of clients based on gender did not account for responses given to the scenario 2.

## Discussion

Lawyers who assist their clients in negotiating a divorce agreement, interpreting and applying the law, are part of an ongoing process of legal intermediation that contributes to the shaping of legal norms (Biland 2023). In doing so, they participate in the implementation of a certain form of gender equality. In recent years, the *formal-egalitarian* interpretation seems to have prevailed in the Swiss legal framework, irrespective of the fact that a large number of women are still not in a position of economic self-sufficiency after a divorce (Kessler 2020, Cottier *et al*. 2022) as in the England and Wales (Douglas 2018).

In the context of a move towards the private ordering of legal issues of separation and divorce (Maclean *et al*. 2015), it proved more important than ever to consider the perspective of lawyers. By analysing the responses of lawyers working in divorce law in Switzerland on two critical topics when negotiating a divorce agreement, we were able to demonstrate that two interpretations of gender equality are in the foreground: the *formal-egalitarian* interpretation based on the principle of autonomy between ex-spouses, and the *compensatory* interpretation based on the principle of compensation of the lasting disadvantages, as conceptualised by Binkert and Wyss (1997). The *compensatory* approach clearly favours spouses who have reduced their activity in the labour market to take care of children – in Switzerland mainly women, as in most Western countries (Maihofer 2014, Le Goff and Levy 2016). The *compensatory* approach is meant to achieve ‘substantive’ justice in family law and values care-work equally with work in the labour market. On average, lawyers participating in our survey mostly attribute a compensatory function to post-marital maintenance with the assumption of a lower hypothetical income, but they are predominantly sceptical about the possibility of compensating for marriage-related disadvantages that arise after the end of the marriage by attributing more than half of the pension assets accrued by the two spouses during marriage to the ex-spouse who is likely to continue working part-time after divorce due to childcare duties. Regarding pensions, a *formal-egalitarian* interpretation, and individual responsibility for savings for old age prevails. Nevertheless, in contrast to England and Wales, where despite the introduction of pension attachment and pension sharing orders in 1996 and 2000, the idea that pensions are personal property still predominates (Woodward and Sefton 2014, Hitchings *et al*. 2023), the principle of equal sharing is well accepted in Switzerland.

It is also worth highlighting that while purely *traditionalist* interpretations were disregarded, mixed interpretations based on gender roles were still chosen. The standardised stories that divorce lawyers actually produce are a ratification of certain norms (Bessière *et al*. 2020) and the conjunction between *formal-egalitarian and traditionalist* interpretations conveys the message that women are still primarily defined by a caregiver role which has to be maintained after divorce and that it is socially acceptable for their living conditions not to be as favourable as those of their male ex-spouses. To take it a step further, the incorporation of these social norms into professional practices contributes to maintaining a semi-traditionalist model with a two-class citizen system, placing caregivers in a lower social position than wage earners. From a life course perspective, gender norms often result in women’s poverty after separation and divorce – as in other Western countries (Fisher and Low 2016, 2018, Hogendoorn et al. 2019, Leopold and Kalmijn 2016) – and a high prevalence of single-parent families relying on social assistance as a rather unexpected consequence of prior life arrangements, which are institutionally validated in the divorce agreement (Widmer and Spini 2017).

It is intriguing to see the lack of consistency among lawyers depending on the issue at hand. It shows that different interpretations of gender equality can coexist and compete at the same time and that lawyers do not follow fundamental principles in their practice but rather strategically adapt such practice to their perceptions of the majority opinion on the specific issue at stake. It points to the nature of the intermediation process (Barlow *et al*. 2017, Pélisse 2019), in which lawyers must act on behalf of alternately female or male clients and skilfully tailor their narratives to the client’s advantage, rather than challenging the normative framework prevailing in law and society, while inadvertently helping to shape it.

When looking closely at the characteristics of lawyers who opt for a *compensatory* solution for post-marital maintenance, the factor of age stands out particularly: older lawyers are more supportive of compensation than younger ones. This indicates a generational shift to more *formal-egalitarian* thinking in the younger generation in line with the new observed trends such as the decline of maintenance payments (Kessler 2020) and the decisions of the Swiss Federal Supreme Court (Cottier *et al*. 2022). Gender is also associated with a *compensatory* solution, but only in the scenario on the division of pension assets. This small association is worth highlighting though, as this ‘textbook’ solution implies an active move to compensate disadvantages but was seldom chosen. Studies on the impact of lawyers’ gender have not been conclusive so far (Bogoch 1997, Mather *et al*. 2001) and, similarly, our findings are limited.

A specialised training in family law also leads to more support for the *compensatory* function. The results of this research therefore indicate the importance of lawyers’ specialisation for the achievement of substantive gender equality in Swiss family law. Finally, the awareness of the existence of gender inequalities proved to be key to promote a *compensatory* solution, while ignoring their existence led to *formal-egalitarian* solutions. Contrarily, statements about treating clients based on gender were not associated with the scenarios, pointing out that lawyers probably adapt their practices to their client’s gender without following overarching principles, as discussed above. Nevertheless, having access to services provided by specialised divorce lawyers is a prerequisite to have a chance to achieve some sort of equality even if not completely, as suggested by Douglas in the English and Welsh context (2018).

Our analysis is based on a scenario approach, which does not capture the full variety of rationales behind divorce lawyers’ interpretations of gender equality, nor reflect their actual practice with their clients. Nevertheless, it is an efficient way of testing their preferences and uncovering the underlying determinants of such preferences. More in-depth qualitative research is needed to understand this intermediation process and the negotiations that take place ‘in the shadow of the law’ (Mnookin and Kornhauser 1978–79).

Overall, our findings argue for specialised training on gender issues in family and divorce law to raise awareness of gender inequalities before and after divorce. Taking structural gender inequalities of men and women better into account in law practices may decrease the impact of institutional doing gender on the life course of women and children. With greater knowledge and understanding of these consequences, divorce lawyers may be better equipped to make full use of the compensatory mechanisms embedded in written law.

## Notes

1. Some authors criticise compulsory court involvement, cf. Fankhauser (2022): n. 19.

2. As noted in the introduction, the main savings of most Swiss divorcees are pension assets, which are governed by special rules designed to protect divorcees from poverty in old age. Matrimonial property law regulates other forms of capital, but only plays a role for wealthier couples, who were not the focus of this study.

3. According to Art. 280 para. 3 SCCP: If the spouses deviate in an agreement from the 50/50 division or if they waive the equal sharing of pension assets in it, the court shall examine ex officio whether adequate old-age and disability provision remains guaranteed.

4. Decision of the Swiss Federal Supreme Court, BGer, 5D_148/2017 of 13 October 2017, FamPra.ch 2018, 220, E. 4.1; Federal Council (2013): 4916.

5. Critically Grütter: 2017: 143, who calls for an interpretation in light of the constitutional requirement of gender equality of Art. 8 para. 3 Swiss Federal Constitution, Art. 14 European Convention on Human Rights and Art. 15 and 16 UN Convention on the Elimination of All Forms of Discrimination Against Women.

6. Decisions by the Swiss Federal Supreme Court BGE 143 III 233 (2017); BGE 137 III 118 (2011).

7. Decision of the Swiss Federal Supreme Court BGE 144 III 481 (2018).

8. To give an impression of the size of the sample, this figure can be put in relation with the 10,774 lawyers who were members of the Swiss Bar Association in 2020, see https://www.sav-fsa.ch/mitgliedschaft.

9. The higher proportion of women in the sample is concordant with the finding by Boni‐Le Goff *et al*. (2020) that women are still overrepresented in the traditionally ‘female’ legal domains, such as family law and labour law.

10. According to the case law of the time, the Federal Supreme Court established the presumption that after ten years or in the case of joint children, a life-shaping marriage was to be affirmed. In the case of life-shaping marriages, the Federal Supreme Court assumed that the trust in the continuation of the marriage or in the continuation of the previous, freely agreed division of tasks was objectively worthy of protection and that Art. 125 para. 1 Swiss Civil Code therefore gave a claim to the continuation of the last common standard of living if the means were sufficient or, if the means were insufficient due to additional costs caused by the divorce, a claim to the same standard of living on both sides (BGE 137 III 102 E. 4.2.1.1; 141 III 465 E. 3.1). In a judgment of 3 November 2020 (BGE 147 III 249), the Federal Supreme Court abandoned this case law.

11. For an in-depth description of the four scenarios see Cottier *et al*. (2022).
